# Benzodiazepine Prescription for Anxiety Disorders Increase the Risk of Substance Use Disorders: A Retrospective Cohort Study

**DOI:** 10.1192/j.eurpsy.2023.716

**Published:** 2023-07-19

**Authors:** C.-F. Sun, Y. Lin, A. S. Pola, A. S. Kablinger, R. L. Trestman

**Affiliations:** ^1^Department of Psychiatry and Behavioral Medicine, Virginia Tech Carilion Clinic School of Medicine, Roanoke, United States; ^2^ Clinical Research Center for Mental Disorders, Shanghai Pudong New Area Mental Health Center, Tongji University; ^3^Department of Psychiatry, Shanghai East Hospital, School of Medicine, Shanghai, China

## Abstract

**Introduction:**

While the role of benzodiazepines (BZDs) has been well established for anxiety and related disorders, there are significant concerns about BZD dependence, withdrawal, and tolerance. There is a lot of ambiguity regarding the potential long-term effects of BZDs on mental health. However, the risk of developing subsequent other substance use disorders is in question.

**Objectives:**

In this electronic medical record (EMR) based retrospective cohort study, the study cohort was defined as patients between the ages of 18 and 65 with anxiety disorders (ICD-10-CM: F40-F48) prescribed with at least one BZD; the control cohort was defined as patients between the ages of 18 and 65 with anxiety disorders (ICD-10-CM: F40-F48) with no BZD prescription during the five-year timeframe examined. We excluded patients with pre-existing substance use disorders (ICD-10-CM: F10-F19), et al.

**Methods:**

We collected data from TriNetX Research database, a real-time international EMR network, from September 2017 to September 2022. Patients in the two cohorts were matched by gender, age, race, ethnicity, and common medical conditions at a 1:1 ratio by propensity scoring and then underwent Kaplan–Meier analysis and association analysis.

**Results:**

A total of 626,754 patients were identified and matched for analysis. Patients in the study cohort were more likely to be female (67.6% vs. 66.7%, p < 0.001), non-Hispanic (65.8% vs. 62.5%, p < 0.001) and white (72.8% vs. 69.1%, p < 0.001). Kaplan–Meier analysis showed the survival probability at the end of the time window was 94.1% for the control cohort and 89.5% for the study cohort (Hazard ratio, 2.20; 95% CI, 2.16-2.25; P < 0.001) in all type of substance use disorders. (Table 1)

**Table 1.** Hazard ratio of substance use disorders difference in BZD cohort versus the control cohort.

	BZD Cohort n (risk%)	Control Cohort n (risk%)	Hazard Ratio (95% Cl)	P value
Substance Use Disorders*	26,569 (4.2)	11,976 (1.9)	2.20 (2.16- 2.25)	<0.001
Sedative/hypnotic/anxiolytic related disorders	656 (0.1)	152 (0.0)	4.26 (3.57- 5.09)	<0.001
Alcohol Related Disorder	5,749 (0.9)	2,064 (0.3)	2.74 (2.61-2.88)	<0.001
Opioid Related Disorder	2,807 (0.4)	815 (0.1)	3.38 (3.13-3.66)	<0.001
Stimulant Related Disorder	1,658 (0.3)	551 (0.1)	2.94 (2.67- 3.24)	<0.001
Cannabis Related Disorder	3,376 (0.5)	970 (0.2)	3.41 (3.17- 3.66)	<0.001

* Substance use disorders was defined as Mental and behavioral disorders due to psychoactive substance use (ICD-10-CM: F10-F19).

**Conclusions:**

Patients with an anxiety disorder who were prescribed BZDs are at higher risk of not only BZD dependence but all types of substance use disorders than a matched cohort not prescribed BZDs. Given this notable association, clinicians should be cautious while prescribing BZDs and inform the patient about the risks associated with their utilization.

**Disclosure of Interest:**

None Declared

